# Innovative Peptide Bioconjugation Chemistry with Radionuclides: Beyond Classical Click Chemistry

**DOI:** 10.3390/ph17101270

**Published:** 2024-09-26

**Authors:** Samantha Leier, Frank Wuest

**Affiliations:** 1 Department of Oncology, Faculty of Medicine and Dentistry, University of Alberta, Edmonton, AB T6G 1Z2, Canada; 2 Faculty of Pharmacy and Pharmaceutical Sciences, University of Alberta, Edmonton, AB T6G 2H1, Canada; 3 Department of Chemistry, Faculty of Science, University of Alberta, Edmonton, AB T6G 2G2, Canada; 4 Cancer Research Institute of Northern Alberta, University of Alberta, Edmonton, AB T6G 2R3, Canada

**Keywords:** click chemistry, radionuclides, radiochemistry, peptides, bioconjugation

## Abstract

**Background**: The incorporation of radionuclides into peptides and larger biomolecules requires efficient and sometimes biorthogonal reaction conditions, to which click chemistry provides a convenient approach. **Methods**: Traditionally, click-based radiolabeling techniques have focused on classical click chemistry, such as copper(I)-catalyzed alkyne-azide [3+2] cycloaddition (CuAAC), strain-promoted azide-alkyne [3+2] cycloaddition (SPAAC), traceless Staudinger ligation, and inverse electron demand Diels–Alder (IEDDA). **Results**: However, newly emerging click-based radiolabeling techniques, including tyrosine-click, sulfo-click, sulfur(VI) fluoride exchange (SuFEx), thiol-ene click, azo coupling, hydrazone formations, oxime formations, and RIKEN click offer valuable alternatives to classical click chemistry. **Conclusions**: This review will discuss the applications of these techniques in peptide radiochemistry.

## 1. Introduction

Radiolabeled peptides have become integral for the functional imaging and therapy of cancer. Favorable pharmacokinetics and improved tumor tissue penetration are just some of the advantages that peptides possess over larger biomolecules, such as antibodies, which can be immunogenic [1]. The recent clinical success of various radiolabeled peptides, such as ^68^Ga-edotreotide (^68^Ga-DOTA-TOC) and ^177^Lu-oxodotreotide (^177^Lu-DOTA-TATE), for the imaging and treatment of neuroendocrine tumors has inspired continued growth and developments in the field of peptide radiochemistry [2,3].

The synthesis of radiolabeled peptides for radiotracer or radiopharmaceutical development relies predominantly on bioconjugation chemistry to enable the incorporation of common radionuclides such as ^18^F (t_1/2_ = 110 min) and radiometals such as ^68^Ga (t_1/2_ = 68 min) and ^64^Cu (t_1/2_ = 12.7 h) [4,5]. This is largely due to the harsh reaction conditions of fluoride chemistry, which prevent direct fluorination of delicate biomolecules or the need for chelators for the incorporation of radiometals. Typically, fluorinated prosthetic groups are employed to facilitate the incorporation of ^18^F into peptides or other biomolecules using various bioconjugation strategies. Similarly, chelators functionalized with reactive chemical groups facilitate the incorporation of radiometals into peptides or biomolecules after attachment via bioconjugation chemistry.

Click chemistry has become an important component of bioconjugation chemistry since its introduction by Sharpless et al. in 1998 [6]. Click chemistry refers to a group of efficient, modular reactions, which naturally favor the formation of heteroatom bonds. As such, the reactions are typically rapid, high yielding, and require only simple purification. When applied to radiochemistry, the efficiency of click reactions provides an ideal approach for the incorporation of short-lived radionuclides into peptides, and the bio-orthogonality of click reactions allows for expansion of application to delicate proteins and biomolecules. Early examples of click reactions in radiochemistry include the copper(I)-catalyzed alkyne-azide [3+2] cycloaddition (CuAAC) for ^18^F-labeling of peptides in radiochemical yields of 54–99% after 10 min, with the only significant limitation being the presence of cytotoxic copper ions, which limits in vivo application [7,8]. Shortly after these initial applications, the strain-promoted azide-alkyne [3+2] cycloaddition (SPAAC) was introduced for the ^18^F-labeling of potential peptide-based PET tracers in radiochemical yields > 95% after 15 min, providing a copper-free approach desirable for in vivo applications where the use of cytotoxic copper is not suitable [9]. Other common copper-free alternatives for ^18^F-labeling include traceless Staudinger ligation and inverse electron demand Diels–Alder (IEDDA) [10,11,12]. Despite these copper-free alternatives, some limitations remain. These include the required modification of naturally occurring amino acids with non-natural reactive groups, such as azides or tetrazines, which may result in additional purification.

Though these classical click reactions continue to play a valuable role in radiochemistry, newly emerging click reactions such as tyrosine-click and sulfo-click offer valuable alternatives to these classical radiolabeling techniques. In addition to these newly emerging click reactions, well-characterized click reactions that have been underutilized in radiochemistry, such as sulfur(VI) fluoride exchange (SuFEx), thiol-ene click, azo coupling, hydrazone formations, oxime formations, and RIKEN click, offer additional alternatives, which may further expand the current scope of radiochemistry. This review describes click-chemistry-based radiolabeling techniques beyond the classical methods. Applications of tyrosine-click, sulfo-click, thiol-ene click, SuFEx, azo coupling, hydrazone formations, and oxime formations in radiochemistry are discussed, with a focus on peptide radiochemistry.

## 2. Tyrosine-Click

**Figure 1:**
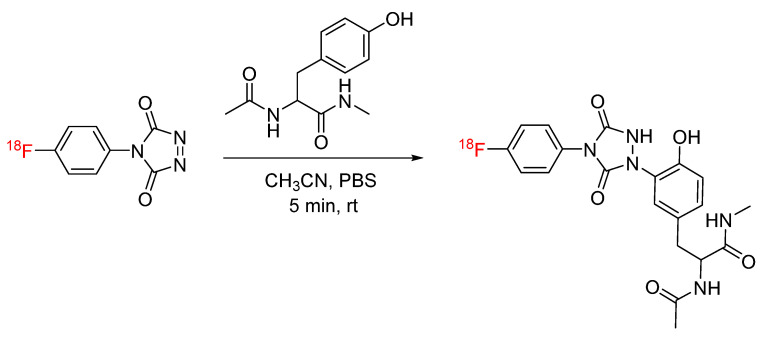
Bioconjugation of [^18^F]F-PTAD with acetyl-L-tyrosine methyl amide via tyrosine-click.

**Figure 2:**
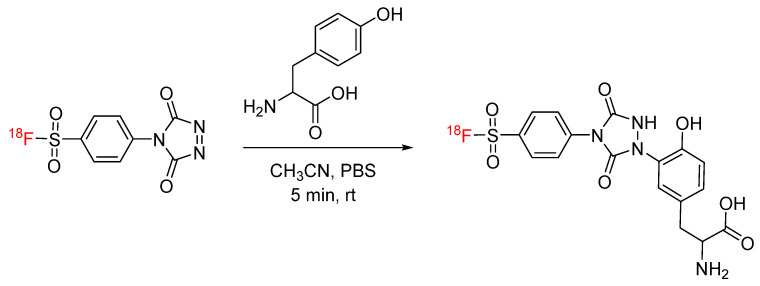
Bioconjugation of [^18^F]FS-PTAD with L-tyrosine via tyrosine-click.

Tyrosine-click chemistry is an emerging bioconjugation strategy in which cyclic diazodicarboxamide derivatives react with the electron-rich phenol ring of tyrosine via an ene-like reaction to produce a heteroatom bond [13,14]. Tyrosine-click chemistry has been applied to radiochemistry through the synthesis of ‘clickable’ prosthetic groups for ^18^F. The first application of tyrosine-click in radiochemistry was reported by Luxen et al. The PTAD-derived prosthetic group [^18^F]4-(4-fluorophenyl)-1,2,4-triazole-3,5-dione ([^18^F]F-PTAD) was synthesized and clicked with tyrosine derivative acetyl-L-tyrosine methyl amide (Figure 1). The reaction was carried out at room temperature for only 5 min, and the clicked product was produced in radiochemical yields of 70% [15]. A similar prosthetic group, 4-(p-[^18^F]fluorosulfonyl)phenyl)-1,2,4-triazoline-3,5-dione ([^18^F]FS-PTAD), was later developed and clicked with L-tyrosine under similar conditions (Figure 2) [16].

The scope of this radiolabeling method is broadened by its short reaction time, which allows for labeling with short-lived radionuclides such as ^18^F, and this emerging bioconjugation strategy may prove to be a valuable addition to peptide radiochemistry. However, the instability of cyclic diazodicarboxamides may present a limitation for application to larger biomolecules [17].

## 3. Sulfo-Click

**Figure 3:**
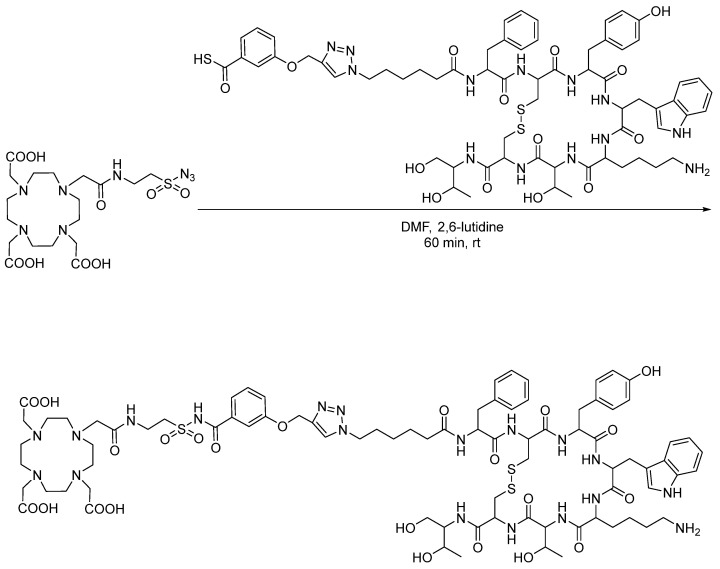
Bioconjugation of DOTA-functionalized sulfonyl azide with octreotide via sulfo-click.

**Figure 4:**
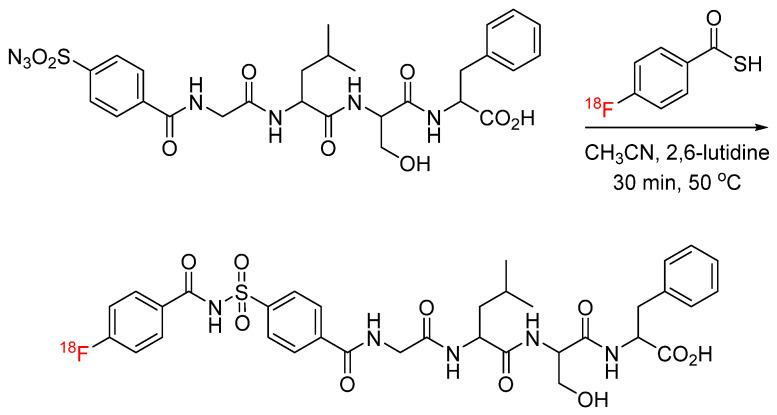
Bioconjugation of in-situ-formed ^18^F-labeled thio acid with sulfonyl-azide-functionalized model peptide via one-pot/three component sulfo-click.

Sulfo-click chemistry is another emerging bioconjugation strategy, in which sulfonyl azides react with thio acids to form an amide bond [18]. Its early applications in radiochemistry included the synthesis of chelator-functionalized sulfonyl azides for conjugation with biomolecules and the subsequent chelation of radiometals. Liskamp et al. synthesized 1,4,7,10-tetraazacyclododecane-1,4,7,10-tetraacetic acid (DOTA)-functionalized sulfonyl azides for conjugation with tyrosine-containing mono-, di-, and tetrameric peptide octreotide (Figure 3) [19]. These DOTA-peptide conjugates were later labeled with ^111^In and used for PET studies with AR42J-tumor-bearing BALB/c mice. The molar activity ranged from 13.6 to 43.3 GBq/µmol for the DOTA-conjugated mono-, di-, and tetrameric octreotide peptides. The binding affinity of octreotide was not significantly impacted by the modification, with IC_50_ values of the corresponding non-radioactive reference compounds ranging from 1.32 to 14.0 nM, and a favorable in vivo profile was observed, with tumor uptake and retention [20,21]. Recently, Wuest et al. reported the first application of sulfo-click chemistry to ^18^F radiochemistry. ^18^F-labeled thio acids generated in situ were clicked with various sulfonyl azides in a one-pot synthesis. Following this initial proof-of-concept experiment, ^18^F-labeled thio acids were clicked with sulfonyl-azide-functionalized celecoxib analogues, as well as sulfonyl-azide-functionalized model peptides (GLSF) (Figure 4). The reaction was carried out under mild conditions, and the ^18^F-labeled celecoxib derivatives and peptides were produced in radiochemical yields of up to 67% and 25%, respectively [22].

While the initial applications of this radiolabeling method used the longer-lived radionuclide ^111^In (t_1/2_ = 2.8 days), the relatively short reaction time allows for the inclusion of shorter-lived radionuclides, such as ^18^F. This radiolabeling method is not just limited to peptides, since mild reaction conditions support future application to larger biomolecules. As well, favorable in vitro and in vivo results for ^18^F-labeled octreotide further support the utility of this radiolabeling method for PET tracer synthesis.

## 4. Sulfur(VI) Fluoride Exchange (SuFEx)

**Figure 5:**
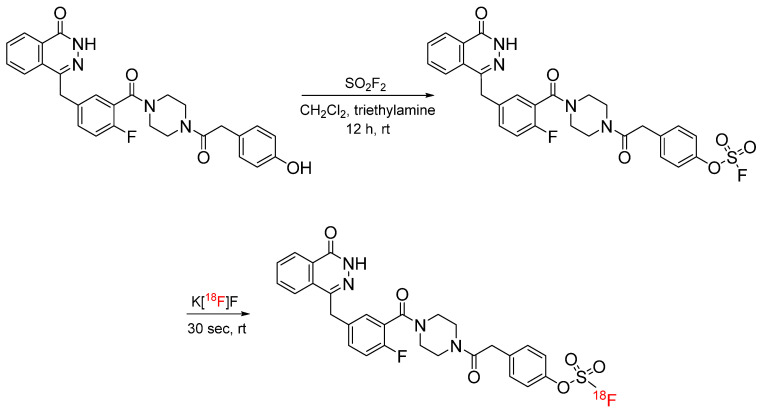
Radiosynthesis of [^18^F]fluorosulfate-functionalized olaparib analog via SuFEx chemistry.

Sulfur(VI) fluoride exchange (SuFEx) is well-characterized chemistry in which sulfur(VI) fluorides react with deprotonated phenols to form a sulfur–oxygen bond and is re-emerging as a bioconjugation strategy with tyrosine [23,24]. Though its application in radiochemistry was previously under developed, recent reports describe the radiosynthesis of [^18^F]fluorosulfate-containing compounds. Zheng et al. synthesized a number of alkyl and aryl [^18^F]fluorosulfates, including an ^18^F-labeled olaparib analog in >95% radiochemical yields with a molar activity of 280 GBq/µmol, for subsequent PET imaging (Figure 5). Unlike typical fluoride exchange, SuFEx with [^18^F]fluoride took place at lower temperatures and did not require lengthy radio-HPLC purification. The IC_50_ of the corresponding ^19^F-labeled olaparib analog was 32.2 nM, demonstrating that there was no impact on the binding affinity. PET studies have demonstrated in vivo stability, as well as tumor uptake, in xenograft mouse models [25]. The procedure has since been optimized to eliminate the need for azeotropic drying and the addition of a base [26]. The optimized procedure has been applied to the radiosynthesis of various ^18^F-labeled radiotracers and radiopharmaceuticals [27,28,29,30]. In an adaptation of this procedure, [^18^F]FSO_2_^+^ was generated in situ to eliminate the need for sulfuryl fluoride gas and cryptand chemistry, which require an anhydrous environment [31].

**Figure 6:**
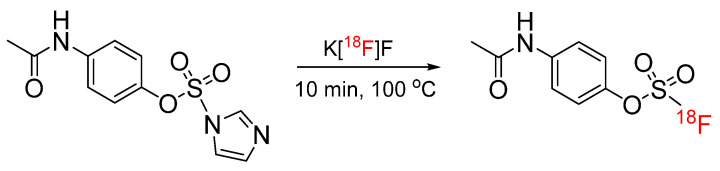
Radiosynthesis of [^18^F]fluorosulfate-functionalized acetaminophen analog via SuFEx chemistry using a fluorosulfuryl imidazolium salt.

[^18^F]fluorosulfates were also synthesized using an alternative method, where sulfuryl fluoride gas was replaced by a fluorosulfuryl imidazolium salt, which was demonstrated by the automated radiosynthesis of an ^18^F-labeled acetaminophen analog (Figure 6) [32]. The ^18^F-labeled acetaminophen analog was afforded 22% radiochemical yield, with a molar activity of 55 GBq/µmol. Overcoming the previous limitations related to the use of toxic sulfuryl fluoride gas, this method is a particularly attractive SuFEx-based approach for ^18^F radiochemistry.

Sulfur(VI)-fluoride-containing compounds provide a promising strategy for radiotracer synthesis. Their dual function as both a precursor for bioconjugation with phenol-containing small molecules or tyrosine-containing peptides, are unique features which provide a platform for ^18^F labeling of small molecules and peptides. Additionally, the rapid and high-yielding reactions that take place under mild conditions and require only simple purification make them ideal for peptide radiochemistry with ^18^F.

## 5. Thiol-Ene Click

**Figure 7:**
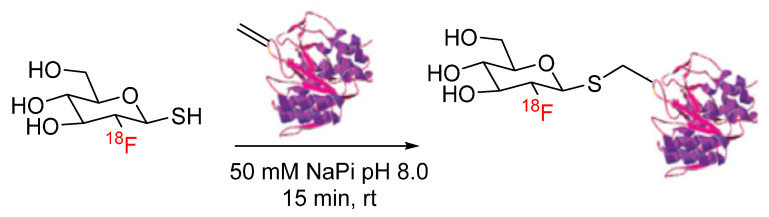
Bioconjugation of [^18^F]FDG-SH with Dha-subtilisin via thiol-ene click.

**Figure 8:**
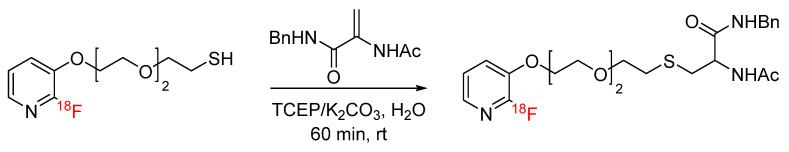
Bioconjugation of [^18^F]fluorothiol prosthetic group with Dha via thiol-ene click.

**Figure 9:**
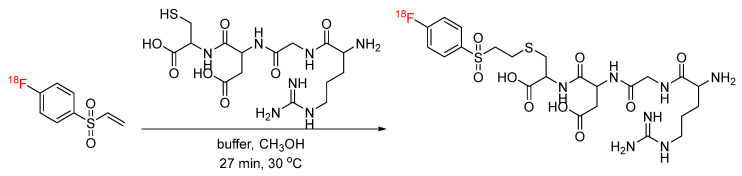
Bioconjugation of [^18^F]FVSB with model peptide via thiol-ene click.

**Figure 10:**
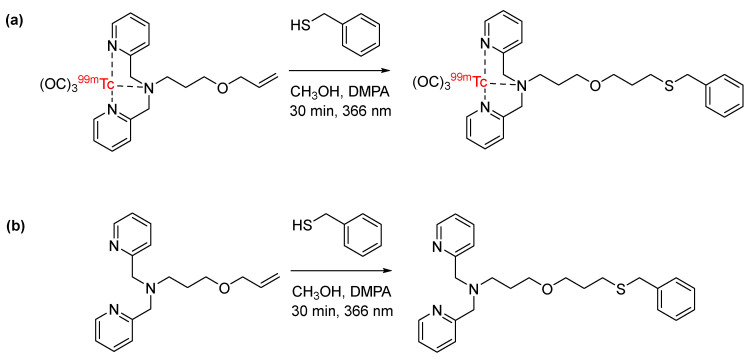
Bioconjugation of an alkene-functionalized DPA derivative with a thiol-containing small molecule, via thiol-ene click, for the incorporation of ^99m^Tc using (**a**) pre-labeling and (**b**) post-labeling strategies.

Thiol-ene click chemistry is a well-characterized bioconjugation strategy in which thiols undergo an anti-Markovnikov addition to an alkene, forming a carbon–sulfur bond [33]. Despite its application in a wide variety of chemistries, its application in radiochemistry had not been explored until recently. Davis et al. reported the synthesis of thiolated [^18^F]fluorosugars for conjugation with dehydroalanine (Dha)-functionalized proteins via thiol-ene click chemistry. Specifically, [^18^F]FDG-SH was synthesized and clicked with dehydroalanine (Dha)-functionalized subtilisin to generate a [^18^F]fluoroglycoprotein (Figure 7). ^18^F-labeled subtilisin was produced in radiochemical yields of 60% [34]. In another approach, [^18^F]fluorothiols were synthesized as prosthetic groups for the incorporation of ^18^F into peptides and proteins. The [^18^F]fluorothiol prosthetic groups were originally introduced by Cuthbertson et al. as alkylating agents for the radiolabeling of chloroacetylated peptides [35]. Based on these initial reports, a [^18^F]fluorothiol prosthetic group has recently been synthesized for use in the thiol-ene click reaction with Dha (Figure 8). The reaction was carried out under reducing conditions, and the [^18^F]fluorothiol-Dha conjugate was produced in radiochemical yields of 37% [36]. This proof-of-concept study also included the fully automated synthesis of the reported [^18^F]fluorothiol. In yet another approach, [^18^F]fluoro-4-(vinylsulfonyl)benzene (FVSB) was synthesized as a prosthetic group for the incorporation of ^18^F into a thiol-containing model peptide (RGDC) (Figure 9). The radiosynthesis of [^18^F]FVSB did not require azeotropic drying of the [^18^F]fluoride, and the final ^18^F-labeled peptide did not require purification, as the radiochemical yields were >96% [37]. Similarly, this approach was applied to the incorporation of ^99m^Tc into a thiol-containing small molecule via alkene-functionalized chelator 2,2′-dipicolylamine (DPA) derivatives. DPA was functionalized with an alkene by the conversion of the alcohol moiety to an ene with allyl bromide in the presence of sodium hydride. Interestingly, this strategy was carried out using both a pre-labeling and a post-labeling approach, which afforded clicked products of the first DPA derivative in 52% and 75% yields, respectively. Pre-labeling and post-labeling afforded the clicked products of the second DPA derivative in 87% and 68% yields, respectively (Figure 10). Note that the chelation of ^99m^Tc with DPA was ≥94% for both strategies [38].

These first applications of thiol-ene click as a radiolabeling method for peptides are promising. The short reaction time makes this method compatible with short-lived radionuclides, and the bio-orthogonality of this method makes it compatible with delicate biomolecules, such as proteins. The availability of [^18^F]FDG at cyclotron facilities is advantageous for thiol-ene click with [^18^F]FDG-SH, and the potential for automation allows for large-scale production, thus expanding the application of this radiolabeling strategy. The near quantitative radiochemical yields of thiol-ene click with [^18^F]FVSB, as well as the elimination of [^18^F]fluoride drying and the elimination of radio-HPLC purification, are also highly advantageous. Additionally, the versatility of this method, including both the pre-labeling and post-labeling approach for incorporation of radiometals, further expands the scope. The only potential limitation of thiol-ene click is the requirement of reducing conditions, which may limit the scope.

## 6. Azo Coupling

**Figure 11:**
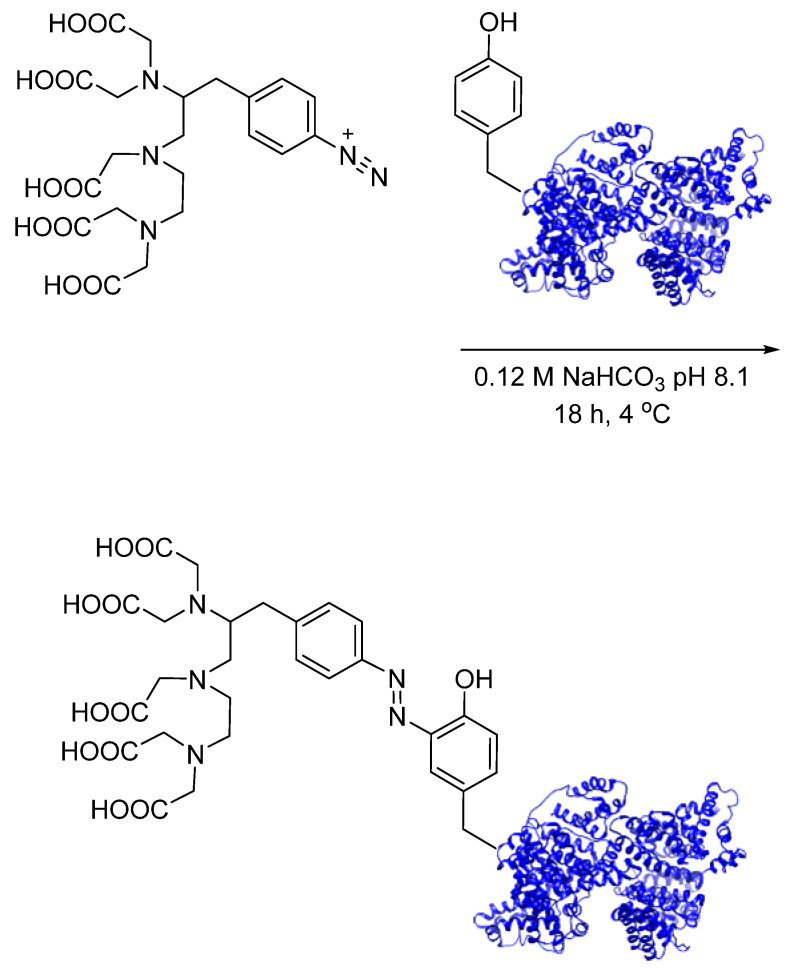
Bioconjugation of EDTA-functionalized diazonium salt with proteins via azo coupling.

**Figure 12:**
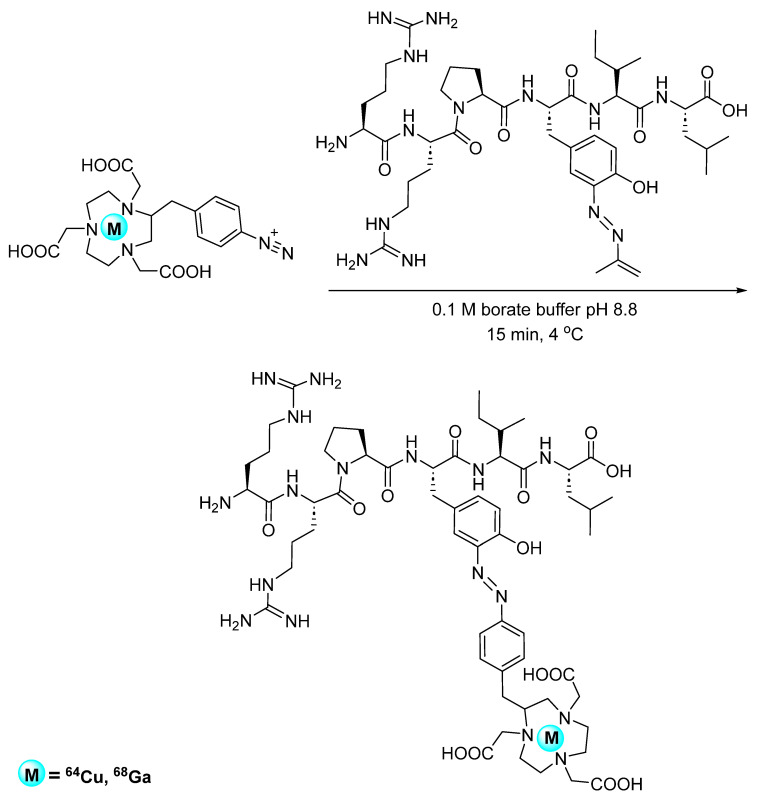
Bioconjugation of ^64^Cu- and ^68^Ga-labeled NOTA-functionalized diazonium salts with neurotensin NT(8–13) via azo coupling.

Azo coupling is among some of the oldest and well-characterized bioconjugation strategies used today. In this strategy, diazonium compounds react with phenols (such as tyrosine) via electrophilic aromatic substitution to produce a heteroatom bond [39]. The early applications in radiochemistry by Diamanti et al. involved the synthesis of diazonium-based chelators for the introduction of radiometals into tyrosine-containing proteins. An ethylenediaminetetraacetic acid (EDTA)-functionalized aryl diazonium salt was coupled with fibrinogen and human serum albumin (HSA) before chelation with ^111^In, and stability was demonstrated in vivo (Figure 11) [40]. Despite these initial efforts, the application of azo coupling in radiochemistry remained relatively unexplored in the years following. This radiolabeling method was revisited for the incorporation of ^111^In and ^125^I into tyrosineamide-containing polymers, with the production of DTPA- and DOTA-functionalized aryl diazonium salts [41]. Wuest et al. further developed and demonstrated the full scope of this radiolabeling method as a PET radiotracer synthesis approach, with the preparation of 1,4,7-triazacyclononane-1,4,7-triacetic acid (NOTA)-functionalized aryl diazonium salts as building blocks for the incorporation of positron-emitting radiometals into tyrosine-containing peptides and proteins. ^64^Cu- and ^68^Ga-labeled NOTA-diazonium salts were prepared in radiochemical yields upwards of 80% using a pre-labeling approach to maintain the reactivity of the diazonium salt. Azo coupling with peptide neurotensin NT(8–13) and protein HSA produced the expected radiolabeled products in only 15 min (Figure 12). A pH-dependent chemoselective trend was also demonstrated, with preferential tyrosine modification above pH 8. IC_50_ values were 0.16 nM and 0.04 nM for ^nat^Cu- and ^nat^Ga-labeled neurotensin NT(8–13), respectively, demonstrating that the binding affinity was not impacted by the modification. PET studies with ^64^Cu-labeled HSA have demonstrated a favorable in vivo profile when compared to other blood pool markers [42]. This technique has since been expanded to include the synthesis of ^64^Cu-labeled GE11-functionalzied micelles for the PET imaging of EGFR-overexpressing tumors [43].

Applications in ^18^F radiochemistry by Pat et al. include the synthesis of ^18^F-labeled diazonium salts for conjugation with tyrosine-containing peptides. ^18^F-fluorobenzenediazonium was originally synthesized for the preparation of ^18^F-thioethers but was later used for azo coupling with tyrosine-containing model peptides (KYS). The ^18^F-labeled peptide was prepared under mild conditions in radiochemical yields of 20% after only 5 min. The radiochemical yield was highly dependent on the amount of peptide used, and lower concentrations resulted in a decreased radiochemical yield and increased side reactions [44,45].

The ability of azo coupling to target naturally occurring amino acids such as tyrosine or histidine, without the modification of the biomolecule, makes it a valuable radiolabeling method for peptides. The high reactivity of diazonium salts and the resulting short reaction times enable use with radionuclides as short-lived as ^68^Ga. Furthermore, mild reaction conditions compatible with proteins expand the scope, and the in vivo stability of linkages contributes to the value of this bioconjugation technique as a radiolabeling method.

## 7. Hydrazone and Oxime Formations

Hydrazone and oxime formations are also among some of the oldest and well-characterized bioconjugation strategies used today. However, slow reaction kinetics have limited their application in radiochemistry, until recent insights led to improved reaction rates, thereby expanding their scope. Hydrazone formations occur when hydrazines react with carbonyl functional groups, such as aldehydes and ketones. Similarly, oxime formations occur when aminooxy groups react with the aforementioned carbonyl groups [46,47,48].

**Figure 13:**
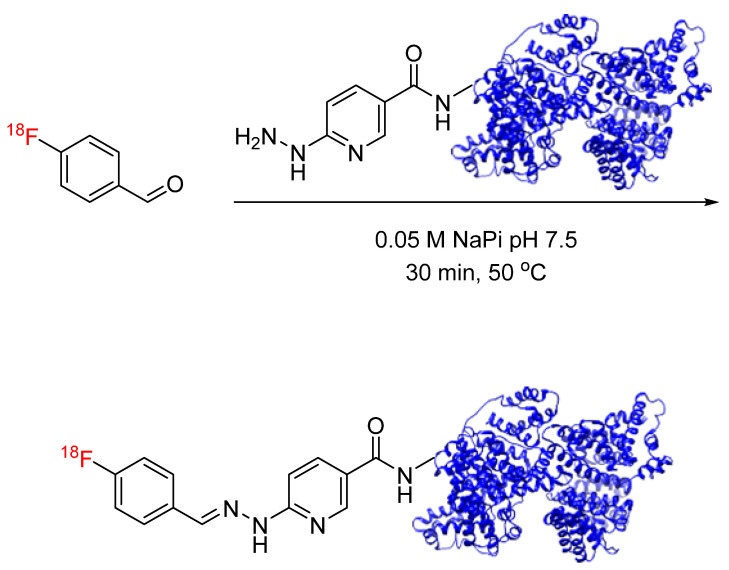
Bioconjugation of [^18^F]fluorobenzaldehyde with HYNIC-HSA via hydrazone formation.

**Figure 14:**
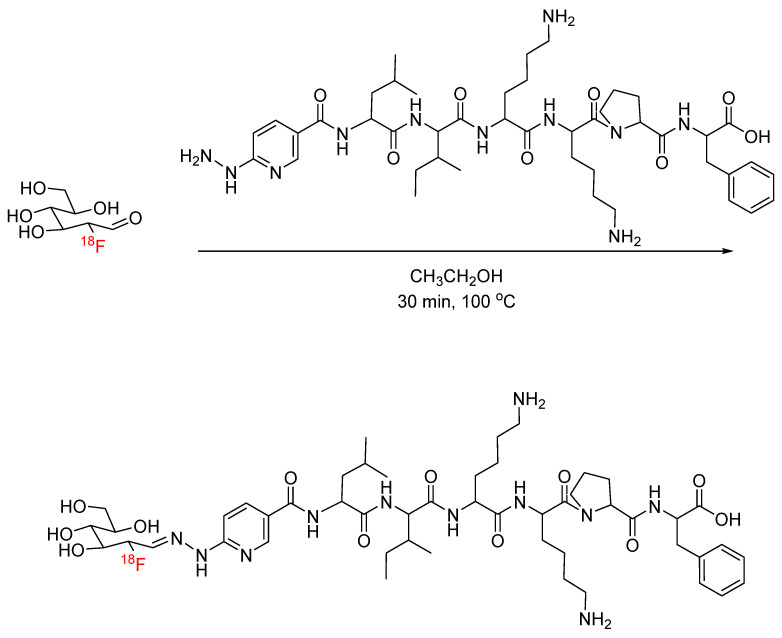
Bioconjugation of acyclic [^18^F]FDG with HYNIC-functionalized peptide via hydrazone formation.

Hydrazone formation has been applied to ^18^F radiochemistry with the synthesis of [^18^F]fluorobenzaldehyde for conjugation with hydrazide-functionalized biomolecules. Lee et al. synthesized [^18^F]fluorobenzaldehyde for conjugation with hydrazinonicotinic-acid-functionalized human serum albumin (HYNIC-HSA) (Figure 13). Radiochemical yields of the ^18^F-labeled HSA ranged from 25 to 90%, and the in vivo stability of the hydrazone linkage was demonstrated through biodistribution studies, where ^18^F-labeled HSA was found primarily in the blood pool 60 min post-injection and exhibited a similar biodistribution profile to that of an approved blood pool marker [49]. This radiolabeling method has since been used by other groups for the ^18^F labeling of peptides (octreotide, substance P, and c(RGDyK)) and small molecule LTB4 antagonist [50,51,52]. Hydrazone formation has also been applied to ^18^F radiochemistry with the synthesis of [^18^F]fluorodeoxyglucose ([^18^F]FDG) for the conjugation of its carbonyl-containing acyclic form with HYNIC-functionalized peptides. Shahhosseini et al. synthesized [^18^F]FDG for conjugation with a HYNIC-functionalized model peptide (LIKKPF) (Figure 14). Mutarotation in an aqueous solution to the acyclic form is critical and was increased by raising the reaction temperatures up to 100 °C [53]. This radiolabeling approach has since been expanded to include the addition of [^18^F]FDG to bifunctional tetrazines as a building block for the production of radiopharmaceuticals [54].

**Figure 15:**
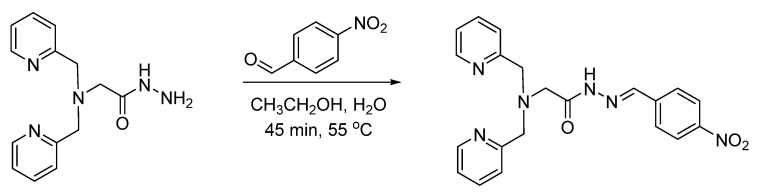
Bioconjugation of DPA-hydrazide with *p*-nitrobenzaldehyde via hydrazone formation.

Despite the application of hydrazone formation in ^18^F radiochemistry, this application in radiochemistry as a whole remains underdeveloped. Benny et al. adapted this bioconjugation strategy for use with radiometals through the synthesis of hydrazide-containing bifunctional chelators for bioconjugation with *p*-nitrobenzaldehyde as an aldehyde-containing model compound. This proof-of-concept study used a hydrazide-functionalized dipicolylamine (DPA) chelator for the incorporation of ^99m^Tc, using both a pre-labeling and a post-labeling approach (Figure 15). Though both approaches produced comparable results, the chelation step was performed at 90 °C, so pre-labeling may be more favorable for protein labeling. They also demonstrated that hydrazone formation was favored over ^99m^Tc coordination with hydrazine in the pre-labeling approach [55]. Recently, the one-pot synthesis of a DOTA-hydrazide was reported by another group [56]. Bioconjugation with aldehyde-containing cellulose nanocrystals (CNC) and the subsequent chelation of ^111^In was then carried out to produce a hydrazone-linked SPECT imaging probe [57].

Overall, hydrazone formation has proven to be a valuable peptide radiolabeling technique. The reported rapid reaction kinetics allow for radiolabeling with short-lived radionuclides, and the stability of the hydrazone bond has also been demonstrated in vivo. The potential for mild reaction conditions allows for the radiolabeling of delicate proteins, although radiochemical yields may be limited since temperatures above 50 °C resulted in the highest radiochemical yields [49]. Similarly, hydrazone formation with [^18^F]FDG requires the temperature-dependent mutarotation of [^18^F]FDG. Temperatures above 80 °C resulted in the highest radiochemical yields, with 100 °C being optimal [53].

**Figure 16:**
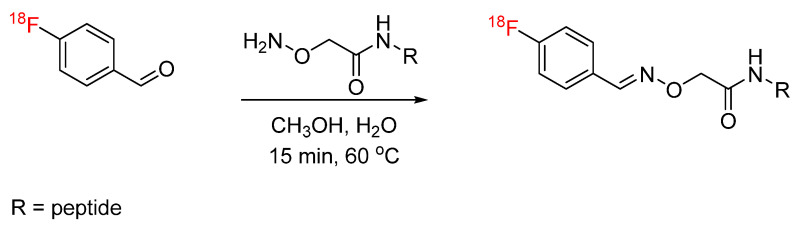
Bioconjugation of [^18^F]fluorobenzaldehyde with aminooxy-functionalized peptides (minigastrin, RGD analogs, and octreotate analogs) via oxime formation.

**Figure 17:**
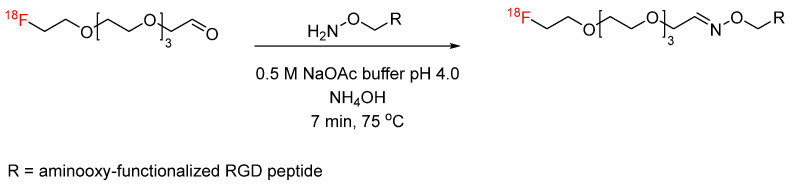
Bioconjugation of ^18^F-(2-{2-[2-(2-fluoroethoxy)ethoxy]ethoxy}ethoxy)-acetaldehyde with aminooxy-functionalized RGD peptide via oxime formation.

**Figure 18:**
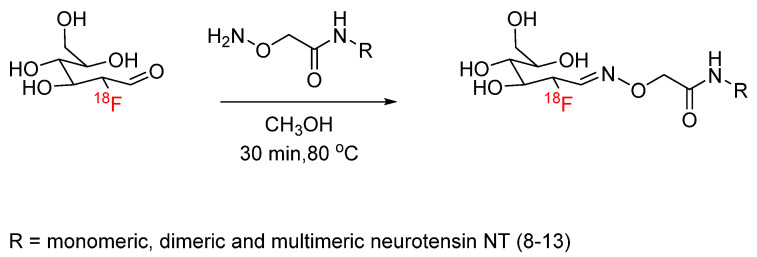
Bioconjugation of [^18^F]FDG with aminooxy-functionalized neurotensin NT(8–13) via oxime formation.

Oxime formation provides an alternative bioconjugation strategy to hydrazone formation. Oxime formation has been applied to ^18^F radiochemistry with the synthesis of [^18^F]fluorobenzaldehyde for conjugation with aminooxy-functionalized biomolecules. Wester et al. synthesized ^18^F-fluorobenzaldehyde for the radiolabeling of aminooxy-functionalized peptides, including minigastrin, RGD, and octreotate analogs, as well as mono-, di-, and tetrameric RGD-containing cyclopeptides (Figure 16) [58,59]. This radiolabeling method was expanded by other groups to include other biomolecules, such as leptin, anti-HER2 dimeric affibody, and chemokine receptor CXCR4 substrate [60,61,62]. Cuthbertson et al. further expanded upon this method with the synthesis of additional aldehyde-based prosthetic groups. ^18^F-(2-{2-[2-(2-fluoroethoxy)ethoxy]ethoxy}ethoxy)-acetaldehyde and ^18^F-4-3-(fluoropropoxy)benzaldehyde were synthesized and conjugated with aminooxy-functionalized RGD (Figure 17). The radiochemical yields ranged from 24% for conjugation with the first prosthetic group to near-quantitative for conjugation with the second prosthetic group. In addition, the ^18^F-(2-{2-[2-(2-fluoroethoxy)ethoxy]ethoxy}ethoxy)-acetaldehyde-RGD conjugate demonstrated a superior in vivo profile when compared to the [^18^F]fluorobenzaldehyde-RGD conjugate [63]. Other aldehyde-based prosthetic groups for oxime formation have since been synthesized by other groups and include ^18^F-5-(1,3- dioxolan-2-yl)-2-(2-(2-(2-fluoroethoxy)ethoxy)ethoxy)pyridine and 2-[^18^F]-fluoro-3-pyridinecarboxaldehyde [64,65]. Another application to ^18^F radiochemistry includes the synthesis of [^18^F]FDG for conjugation with aminooxy-functionalized peptides. Wuest et al. used [^18^F]FDG for conjugation with aminooxy-functionalized neurotensin NT(8–13) (Figure 18). The conjugates were obtained in radiochemical yields of up to 88% [66]. Similarly, this radiolabeling approach has also been used for the addition of [^18^F]FDG to bifunctional tetrazines as a building block for the production of radiopharmaceuticals [67]. In the synthesis of new potential PET tracers, ^18^F-labeled RGD and glyglycine were first synthesized as model tracers in the initial proof-of-concept PET studies [68,69]. ^18^F-labeled phosphatidyl-serine-targeting peptide (LIKKPF) and glioma-targeting peptide (RRL) were later synthesized as PET tracers for the imaging of apoptosis and glioma, respectively. In both cases, the uptake into the target tissue was significant [70,71,72]. This method was further expanded by Zanda et al. to include [^18^F]fluorodeoxyribose ([^18^F]FDR) for bioconjugation with cyclic RGD peptides (c(RGDfK) and c(RGDfC)) through oxime formation [73]. [^18^F]FDR was used by another group for bioconjugation with tetrazine derivatives, which are not stable under harsh fluorination conditions, to produce an ^18^F-labeled glycosylated tetrazine [74]. The use of FDR rather than FDG was shown to produce significantly higher yields in non-radioactive conjugation experiments with c(RGDfC)) [71]. The conversion to the glycosylated peptide product was determined by HPLC to be >80% for FDR and <30% for FDG. This result was partially attributed to the thermodynamic stability of the 6-membered ring of FDG, which favors the cyclic form of the sugar rather than the aldehydic form required for conjugation.

Oxime formation provides a rapid, high yielding radiolabeling technique ideal for radiosynthesis peptide-based PET tracers and radiopharmaceuticals. However, higher temperatures (80 °C), when used with [^18^F]FDG, present a challenge for protein labeling where temperature must be limited [58].

## 8. RIKEN Click

**Figure 19:**
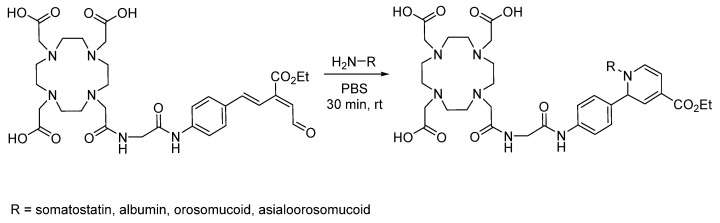
Bioconjugation of aldehyde-functionalized DOTA with somatostatin via RIKEN click.

RIKEN click describes chemistry in which unsaturated aldehydes react with amines via 6π-azaelectrocyclization [75]. Applications in radiochemistry include the synthesis of aldehyde-functionalized chelators for conjugation with peptides and proteins. Fukase et al. synthesized an aldehyde-functionalized DOTA chelator for conjugation with somatostatin, albumin, orosomucoid, and asialoorosomucoid for subsequent ^68^Ga chelation (Figure 19) [76]. DOTA-conjugated somatostatin showed the expected results in vivo up to 4 h post-injection, demonstrating the stability of the conjugate and the potential of this click reaction for radiochemistry. They later expanded the scope to include lysine-based dendrimer-type N-glycan clusters [77]. Further optimization included a pre-labeling strategy for improved bio-orthogonality, avoiding the exposure of biomolecules to the harsh chelation conditions of ^68^Ga with DOTA. Additionally, aldehyde-functionalized DOTA pre-labeled with ^68^Ga was clicked with cyclic RDGyK peptide in 71% radiochemical yield and used for PET imaging of α_V_β_3_ integrin-overexpressing U87MG tumor xenografts in BALB/c mice [78]. PET imaging showed a favorable in vivo profile, with significant tumor uptake being observed 60 min post-injection, demonstrating the feasibility of this bioconjugation strategy for radiotracer synthesis.

RIKEN click provides an efficient method for chelator attachment under mild conditions. The short reaction time is ideal for short-lived radionuclides, such as ^68^Ga. The ability to directly label naturally occurring lysine residues without modification makes this strategy desirable for PET tracer synthesis.

## 9. Conclusions

Click-chemistry-based radiolabeling techniques beyond the classical methods have been discussed, with a focus on applications in peptide radiochemistry. These include newly emerging click reactions such as tyrosine-click and sulfo-click, as well as well-characterized click reactions, which have not been widely applied to radiochemistry, such as SuFEx, thiol-ene click, azo coupling, hydrazone formations, oxime formations, and RIKEN click. The described techniques not only contribute to and expand upon the current peptide radiolabeling methodologies but may also offer superior bioconjugation strategies for radiochemistry with peptides and even proteins.

Tyrosine-click, azo coupling, SuFEx chemistry, and RIKEN click are advantageous, since they target naturally occurring amino acid residues and do not require the modification of the peptide. SuFEx also provides an improved radiofluorination technique with [^18^F]fluoride. However, it should be noted that amino acids, such as tyrosine, are often in low abundance and may be located in the peptide or protein core. While this may allow for site-selective conjugation when accessible, post-modification binding affinity is also an important consideration. Post-modification binding affinity was demonstrated for a small molecule and a peptide, with IC_50_ values in the nanomolar range [25,42]. On the other hand, thiol-ene click, hydrazone formation, and oxime formation techniques can utilize common precursors such as [^18^F]FDG and [^18^F]fluorobenzaldehyde, which are typically available at cyclotron facilities via automated synthesis. In addition, thiol-ene click with [^18^F]fluoride offers superior radiofluorination chemistry with the elimination of [^18^F]fluoride drying and the elimination of radio-HPLC purification.

These emerging radiolabeling techniques provide improved and efficient strategies for the radiosynthesis of peptide-based tracers and pharmaceuticals. Although future work with newly emerging tyrosine-click and sulfo-click is needed in order to expand upon these initial proof-of-concept studies, the current results are promising. The remaining click reactions, though well characterized, are still emerging in radiochemistry, and future applications may enable the synthesis of a variety of new peptide-based radiotracers and radiopharmaceuticals.
